# RACIAL-ETHNIC DISPARITIES IN HEALTHCARE SATISFACTION

**DOI:** 10.1093/geroni/igz038.3021

**Published:** 2019-11-08

**Authors:** Collin Mueller, Heather Farmer

**Affiliations:** Duke University, Durham, North Carolina, United States

## Abstract

This paper explores how perceptions of unfair treatment shape healthcare satisfaction across race/ethnicity. We investigate the overall impact of life course exposure to healthcare discrimination on current healthcare satisfaction across race/ethnicity among a sample of midlife and older Black, Latinx, and White Americans age 50+ in the Health and Retirement Study. We then test whether everyday healthcare discrimination mediates the impact of major medical discrimination on healthcare satisfaction, controlling for sociodemographic factors, mental and physical health characteristics, functional status, life course stress exposure, and lifetime and everyday discrimination in contexts beyond healthcare settings. Black Americans had poorer healthcare satisfaction than White Americans. Everyday discrimination in healthcare settings mediated a modest amount of the relationship between lifetime healthcare discrimination and healthcare satisfaction, and this association varied in strength across White, Black, and Latinx Americans. Results underscore the need for future work identifying and addressing mechanisms shaping healthcare satisfaction.

